# Fusion with extracellular domain of cytotoxic T-lymphocyte-associated-antigen 4 leads to enhancement of immunogenicity of Hantaan virus DNA vaccines in C57BL/6 mice

**DOI:** 10.1186/1743-422X-8-448

**Published:** 2011-09-23

**Authors:** Feng Liu, Mifang Liang, Shouchun Cao, Qinzhi Liu, Quanfu Zhang, Chuan Li, Shuo Zhang, Shiwen Wang, Dexin Li

**Affiliations:** 1 Laboratory for Viral Hemorrhagic Fever, National Institute for Viral Disease Control and Prevention, China CDC 100 Ying Xin Jie, Xuan Wu Qu, Beijing 100052, China

## Abstract

**Background:**

Hantaan virus (HTNV) is the causative agent of the most severe form of a rodent-borne disease known as hemorrhagic fever with renal syndrome (HFRS). A safe and effective HTNV vaccine is needed. Vaccination with DNA constructs expressing fused antigen with bioactive factors, has shown promising improvement of immunogenicity for viral agents in animal models, but the effect of fusion strategy on HTNV DNA vaccine has not been investigated.

**Results:**

DNA plasmids encoding the HTNV nucleocapsid protein (N) and glycoprotein (Gn and Gc) in fusion to the extracellular domain of cytotoxic T-lymphocyte-associated-antigen 4 (eCTLA-4) targeting to antigen presenting cells (APCs) were constructed. Intramuscular immunization of mice with plasmids expressing eCTLA-4-HTNV-N/GP fusion proteins leads to a significant enhancement of the specific antibody response as well as cytotoxic T-lymphocyte (CTL) response in C57BL/6 mice. Moreover, this effect could be further augmented when co-administered with CpG motifs.

**Conclusions:**

Modification of viral antigen in fusion to bioactive factor will be promising to confer efficient antigen presentation and improve the potency of DNA vaccine in mice.

## Background

Hantaan virus (HTNV) (Bunyaviridae family, Hantavirus genus) is the causative agent of the most severe form of a rodent-borne disease known as hemorrhagic fever with renal syndrome (HFRS). Other hantaviruses that are known to cause HFRS include Seoul virus (SEOV), Dobrava virus (DOBV) and Puumala virus (PUUV), which cause disease in Asia, Europe, Scandinavia, and western Russia respectively [1]. In addition, a few hantaviruses have been identified to associate with outbreaks of a highly lethal disease, hantavirus pulmonary syndrome (HPS), in the Americas [2]. Since hantaviruses can cause epidemics with high morbidity, and currently there is no proven therapy for hantaviral disease, a safe and effective vaccine(s) against hantaviruses infection is necessary. HTNV causes the most severe form of HFRS and around 150,000 cases of HFRS are reported worldwide annually, with the majority of HFRS occurring in Asia [3].

Hantaviruses are enveloped, negative strands RNA viruses consisting of three single RNA segments designated S (small), M (medium), and L (large), which encode the nucleocapsid (N) protein, envelope glycoproteins (Gn and Gc), and the RNA polymerase respectively [4]. As a key surface antigen, glycoproteins (Gn and Gc) bear the epitopes which could elicit neutralizing antibodies against hantavirus infection [5]. N-specific antibodies are neither neutralizing nor protective, but may play a role through cellular immune response [5].

Immunization with DNA vaccines encoding antigen has been used to induce both humoral and cellular immune responses and holds potential for developing vaccines to a variety of viral antigens. Application of DNA vaccine to hantavirus was also promising and previously explored. DNA vaccination with a plasmid containing the SEOV M segment elicited neutralizing antibody responses in mice and hamsters as well as a certain level of cross-protection against HTNV [6,7]. A HTNV M gene-based DNA vaccine conferred good protection against infection in hamster model and elicited high levels of neutralizing antibodies in Rhesus monkeys [8]. However, there are still concerns about the potency of DNA vaccines, like a low level of protein expression after DNA immunization.

One of interesting approaches, to improve the potency of DNA vaccine, is to fuse a bioactive domain, like cytotoxic-T-lymphocyte-associated protein 4 (CTLA-4), to viral antigens [9]. CTLA-4 consists of extracellular domain, transmembrane domain and cytoplasmic domain. As an inhibitory costimulatory molecule, CTLA-4 normally plays a key role to downmodulate T-cell activation by interaction with its ligand, B7 on antigen presenting cells (APCs) [10,11]. However, the affinity of CTLA-4 to the shared ligands, B7 is 10-20 times higher than that of its counterpart, CD28 which provides a costimulatory signal to APCs [10]. Recently, Axel et al demonstrated that without the cytoplasmic domain of CTLA-4, the extracellular domain of CTLA-4 (eCTLA-4) alone can enhance TCR activation instead of inhibitory function in the full-length form [12]. Lu et al has observed an enhancement of specific immune response in mice and woodchuck models conferred by eCTLA4 fused with woodchuck hepatitis virus nucleoprotein [13]. In addition, adjuvant effects of CpG motifs have been shown to enhance antigen-specific immune responses to protein vaccine in mice and human [14,15]. While the effects of CpG motifs co-delivery on immune responses to DNA vaccination in mice are diverse [16-18].

In this study, we first report to generate recombinant HTNV DNA vaccine plasmids encoding HTNV N or GP fused to eCTLA4, and evaluated their immunogenicity in C57BL/6 mice as well as the strategy of co-delivery with CpG motifs. Our results indicated that eCTLA4 fusion strategy could enhance specific antibody response and cellular immune response in mice generated by HTNV DNA vaccine. This adjuvant effect could be further augmented when co-delivery with CpG motifs.

## Materials and Methods

### Cells and viruses

The 293T, Vero E6 cells and Baby hamster kidney cell (BHK) cells were purchased from ATCC (ATCC number: CRL-1586) and cultured in Dulbecco modified Eagle medium (DMEM) supplemented with 10% heat-inactivated fetal calf serum, 100 U of penicillin, and 100 μg of streptomycin per ml at 37 with 5% CO_2_. HTNV strain 84Fli, isolated from liver of a fatal fetus in China [19], were grown in Vero E6 cells as previously described [20,21].

### Construction of plasmids for DNA vaccination

Plasmids expressing HTNV strain 84Fli N protein (pcDNA3/S) and glycoproteins (Gn and Gc, pcDNA3/M) were constructed previously [22] with pcDNA3 vector (Invitrogen, Karlsruhe, Germany). A plasmid, pCTLA-4-C expressing eCTLA-4-antigen fusion protein, was a kind gift from Prof. Mengji Lu [13]. pCTLA-4-C was constructed on pcDNA3 vector background with antigen fragment inserted downstream of eCTLA-4 between EcoRV and Xhol (BioLabs, USA) restriction sites. The S and M fragments respectively encoding N protein and glycoproteins (Gn and Gc) were amplified by RT-PCR with the following primers containing restriction enzyme sites (EcoRV and Xhol): S forward: 5'-GGA TAT CAT GGC AAC TAT GGA GGA A-3'; S reverse: 5'-GCA CTC GAG TTA TAG TTT TAA AGG CTC TTG GTT GG-3', M forward: 5'-GGA TAT CAT GGG GGT ATG GAA GTG GCT AGT A-3'; M reverse: 5'-GCA CTC GAG CTA TGA CTT TTT ATG CTT TCT TAC AGG-3'. The amplified fragments of S and M were digested with EcoRV and Xhol and then respectively inserted into the corresponding site of pCTLA-4-C predigested with EcoRV and Xhol to generate pcDNA3/eCTLA4-S and pcDNA3/eCTLA4-M. Insertion of correct nucleotide sequence was verified by sequencing. DNA plasmids were prepared with the Giga plasmid purification kit (QIAGEN, Germany), and then dissolved in phosphate-buffered saline (PBS) in a final concentration of 1 mg/ml.

The S and eCTLA4-S fragments were also further cloned into pET30a vector (Merck, Darmstadt, Germany) respectively to generate pET30a/S and pET30a/eCTLA-S for the identification of expression of HTNV N and eCTLA4-N fusion protein in prokaryotic system induced by isopropyl-beta-D-thiogalactoside (IPTG).

### Expression and Identification of eCTLA4-HTNV N and eCTLA4-GP fusion proteins

Prokaryotic expression of HTNV N and eCTLA4-NP fusion protein was analyzed by sodium dodecyl sulfate polyacrylamide gel electrophoresis (SDS-PAGE) as described previously [23]. 293T and BHK cell lines were used for transfection experiment. Transient transfection was performed by using Lipofectamine 2000 (Invitrogen, USA) according to the manufacture's instructions. Transient expression of fusion protein, eCTLA4-N and eCTLA4-GP (Gn and Gc) was verified by western-blot or immuno-fluorescence assay (IFA) respectively as described previously [23].

### Immunization of mice with HTNV recombinant DNA plasmid

Female 6-8 weeks old C57BL/6C mice (H-2K^b^) were housed in the facility of Chinese Academy of Medical Sciences Breeding Laboratories under specific-pathogen-free conditions. Mice were pretreated by intramuscular injection of 100 μl 0.25% bupivacaine [24] in quadriceps with 50 μl in each side. 24 hours later, groups of mice were injected intramuscularly (i.m.) three times at one week interval with 100 μg of DNA plasmids in the presence or absence of 10 μg of CpG1826 motifs [25] (Sangon Biotech, Shanghai, China). DNA plasmids expressing HTNV N (or eCTLA4-N) and HTNV GP (or eCTLA4-GP) were mixed equally with 50 μg of each. A group of mice was injected i.m. with either 100 μg of pcDNA3 vector or 100 μl PBS alone as a negative control. Sera of 5 mice per group for serological assay were collected before each immunization and one week after third immunization, and for cellular immune response assay were collected one week after second immunization.

### Serologic assays

HTNV N-specific IgG antibodies in mice sera were determined, by enzyme-linked immunosorbent assay (ELISA) in [26,27]. 96-well microtiter plates (Costar, USA) coated with 100 μl of purified recombinant N protein of HTNV strain A9 at a concentration of 1 μg/ml as described previously [23]. Hantaan virus glycoproteins specific IgG antibodies were evaluated by IFA using insect Sf9 cells infected with a recombinant baculovirus expressing the glycoproteins (Gn and Gc) of HTNV train A9 [23]. Titers of neutralizing antibody were also determined by microneutralization (MN) assay as previously described [23].

### Enzyme Linked Immunospot (ELISPOT) Assay

All antibodies and reagents used in cytokine ELISPOT assays were purchased from BD/Pharmingen (San Diego, CA, USA). BD™ ELISPOT plates (BD, USA) were coated with 100 μl of anti-mouse IFN-γ Ab (5 μg/ml in Coating Buffer) at 4°C overnight. The plates were then blocked with Blocking Solution (RPMI1640) for 2 h at room temperature. 100 μl freshly isolated splenocytes (5 × 10^5 ^cells) were added into each wells and stimulated with a synthesized peptide (HTNV N protein 221-228: SVIGFLAL) at 10 μg/ml, or positive stimulators TPA (20 ng/ml) and Ionomycin (1 μg/ml). The plates were incubated for 24 h at 37°C with 5% CO_2_. Development and counting of cytokine ELISPOTs were performed following the manufacturer's procedures. Spots were counted using an ELISPOT reader system (ImmunoSpot^® ^Analyzer, USA).

### Intracellular Cytokine Staining (ICS) Flow Cytometer

For the analysis of intracellular IFN-γ cytokine, freshly isolated splenocytes (5 × 10^6 ^cells) were incubated for 5 h at 37°C in RPMI containing 10% FBS and 10 μg/ml peptides (HTNV N protein 221-228: SVIGFLAL), or a positive stimulator brefeldin A (Sigma, USA) at 10 μg/ml. After being stained with FITC-conjugated anti-CD8 antibody and PE-cy5 conjugated anti-CD3 antibody (eBioscience, USA), cells were fixed with 4% paraformaldehyde in PBS for 15 min, and then were permeabilized with 0.5% saponin (Sigma, USA) in PBS for 10 min. Finally, cells were stained with PE-conjugated anti-mouse IFN-γ McAb. All the procedures of antibody staining were performed at room temperature for 15 min. Cell samples were then analyzed with an Epics-MCL Cytometer (Beckman Coulter, USA), and the data were collected with EXPO32 ADC XL 4 Color Software.

### Statistical analysis

Statistical significance of the data was determined by using Student's t test or ANOVA of the SPSS 10.0 software. The antibody titers were log_10 _transformed to get a normal distribution before statistical analysis. A *P *value of < 0.05 was considered significant.

### Ethical approval

According to the medical research regulation of Ministry of Health, China, this study was approved by the ethics committee of China CDC, which uses international guidelines to ensure confidentiality, anonymity, and informed consent. Informed consent was obtained from all study participants.

## Results

### Expression and identification of eCTLA4-N/GP fusion proteins

**Figure 1:**
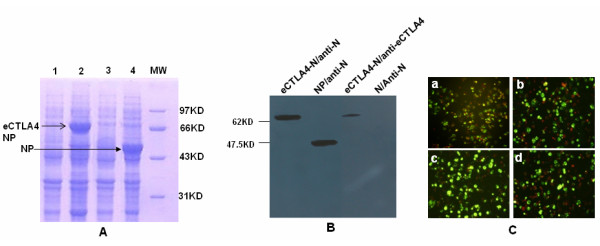
**Expression and identification of fusion proteins of eCTLA4-HTNV N or eCTLA4-HTNV glycoproteins**. (A) SDS-PAGE analysis of eCTLA4-HTNV N fusion protein in supernatant (Lane 1) and cell lysates (Lane 2) of *E.coli*; N protein without eCTLA (Lane 3 and 4); (B) Western-blot analysis of eCTLA4-HTNV N fusion protein from 293T cells transfected with DNA constructs, detected by either N-specific monoclonal antibody L13F3 (eCTLA4-N/anti-N) or anti-mouse eCTLA-4 antibody (eCTLA4-N/anti-eCTLA4). As a control, the HTNV N protein without fusion with eCTLA-4 was also detected by monoclonal antibody L13F3 (NP/anti-N). (C) Immuno-fluorescence assay of transient expression of eCTLA4-HTNV GP detected with HTNV Gc specific monoclonal antibody Y22 or anti-mouse eCTLA-4 antibody (C-a and b); or transient expression of eCTLA4-HTNV N fusion proteins detected with L13F3 and anti-mouse eCTLA-4 antibody (C-c and d).

The expressing of eCTLA4-N fusion protein was firstly determined in prokaryotic system. Supernatant and cell lysates of *E.coli *DH5α transformed with pET30a/eCTLA-S or pET30a/S plasmid were analyzed by SDS-PAGE (Figure 1A). Neither eCTLA4-N fusion protein nor HTNV N was expressed into supernatant (Figure 1A, lane 1 and lane 3). Instead, eCTLA4-N fusion protein was shown in cell lysate (Figure 1A, lane 2) with about 66KD of molecular weight (MW). HTNV N was consistently seen in 50KD of MW (Figure 1A, lane 4) which matches previous result [23]. The expression of eCTLA4-N fusion protein was further examined by western-blot from 293T cells transiently transfected with pcDNA3/eCTLA4-S. As shown in Figure 1B, eCTLA4-N fusion protein was detected by either N-specific monoclonal antibody of L13F3 [28,29] (eCTLA4-N/anti-N) or anti-mouse eCTLA-4 (eBioscience, USA) (eCTLA4-N/anti-eCTLA4). As a control, the HTNV N protein without fusion with eCTLA-4 was also detected by monoclonal antibody L13F3 (NP/anti-N).

IFA was also used to verify the expression of eCTLA4-N and eCTLA4-GP fusion proteins as described in Methods. BHK cells were transiently transfected with pcDNA3/eCTLA4-M or pcDNA3/eCTLA4-S construct. The expression of eCTLA4-GP or eCTLA4-N fusion protein was detected with Gc- specific antibody (Y22) or N-specific antibody (L13F3) [28,29] respectively as demonstrated in (Figure 1C, a and 1c). Furthermore, eCTLA4-GP and eCTLA4-N fusion proteins could also be captured by monoclonal antibody of anti-mouse eCTLA-4 (Figure 1C-b and 1d).

### Antibody responses to HTNV N and GP induced in mice following immunization with plasmids expressing eCTLA4-N/GP fusion protein

**Figure 2:**
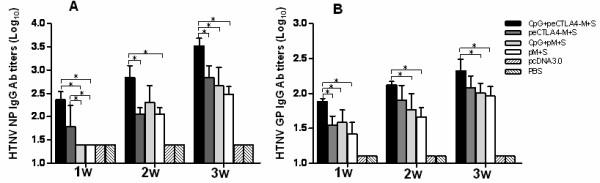
**Kinetics of anti-HTNV N and anti-HTNV GP IgG antibody responses**. Mice were intramuscularly immunized with 100 μg of DNA constructs with or without CpG motifs on day 0, day 7 and day 4 and bled before each immunization. HTNV N and GP specific IgG antibody responses were evaluated by ELISA (A) and IFA (B) respectively. Antibody endpoint was reciprocal of the highest dilution of serum that conferred optical density above cutoff or positive fluorescence signal. *p < 0.05 between two groups denoted by the capped line.

To evaluate whether eCTLA4 fusion strategy could enhance immunogenicity on HTNV DNA vaccine, C57 mice were immunized with DNA plasmids expressing HTNV N and GP, or eCTLA4-N and GP fusion proteins with or without 10 μg of CpG1826 motifs. The antibody immune response to HTNV N or GP was determined by N-specific ELISA or IFA assays. (Figure 2) The levels of N protein-specific IgG were found to be substantially induced one week after first immunization in mice that received pcDNA3/eCTLA4-S+M DNA plasmids alone or with CpG1826 (Figure 2A), and significantly higher than that of mice receiving pcDNA3/S+M DNA plasmids alone or with CpG1826. One week after second injection, mice immunized with pcDNA3/eCTLA4-S+M plasmids plus CpG1826 showed significantly higher N protein-specific IgG antibody titers compared to groups of mice that received pcDNA3/eCTLA4-S+M or pcDNA3/S+M DNA plasmids alone (p < 0.05), and about 3.5-fold higher than that of mice receiving pcDNA3/S+M DNA plasmids plus CpG1826 though not achieved statistic significance. After two boosts, all mice that received HTNV DNA vaccine plasmids had substantial increase of N protein-specific IgG antibody titers. DNA plasmids expressing eCTLA4-N and GP fusion proteins, combined with CpG1826, elicited the highest N protein-specific IgG antibody titers one week after third immunization compared to all the other groups (p < 0.05). In addition, we also observed that the magnitude of glycoprotein specific IgG antibody was significantly improved by vaccination with DNA plasmids expressing eCTLA4-N and GP fusion proteins, especially when combined with CpG1826 (Figure 2B). No eCTLA-4-specific antibodies were detected in sera of mice receiving DNA plasmids expressing fusion proteins (data not shown), which is consistent with the results of Lu et al [13]. These results indicate that eCTLA4 fusion strategy and CpG motif could improve the immunogenicity of HTNV DNA vaccine.

### Neutralization activity

Neutralizing antibodies, which conferring protective immunity induced by DNA vaccine plasmids against hantavirus were evaluated by microneutralization assays. As shown in Table 1, pre-immune sera from all the groups exhibited no neutralizing activity. In contrast, immune sera collected 21 days after the first immunization with 100 μg of HTNV DNA vaccine plasmids in the presence or absence of CpG motifs showed neutralizing antibody titers of 8 to 32 (reciprocal of the highest dilution exhibiting 50% neutralization) against HTNV strain 84Fli. Immunization with pcDNA3 vector didn't elicit any neutralizing antibody. Groups of mice receiving pcDNA3/eCTLA4-S+M plus CpG motifs, pcDNA3/eCTLA4-S+M alone, or pcDNA3/S+M plus CpG motifs, all achieved MN titers of ≧ 16. Only three of mice receiving pcDNA3/S+M alone could achieve MN titers of 16. The mean MN titer in mice vaccinated by pcDNA3/eCTLA4-S+M plus CpG motifs or pcDNA3/eCTLA4-S+M alone was significantly higher than that of mice immunized with pcDNA3/S+M alone (*p *< 0.05). These results indicated that eCTLA4 fusion strategy combine with CpG motif could induce better magnitude of neutralizing antibodies in mice against HTNV infection.

**Table 1:** Neutralizing antibody responses against HTNV in mice 21 days after 1^st ^vaccination

		Titers for 50% neutralization	
		
Vaccination groups	**Mouse no**.	Day 0	Day 21
CpG+pcDNA3/eCTLA4-S+M	1	< 8	16
	2	< 8	32
	3	< 8	32
	4	< 8	16
	5	< 8	16
			
pcDNA3/eCTLA4-S+M	1	< 8	16
	2	< 8	16
	3	< 8	16
	4	< 8	16
	5	< 8	32
			
CpG+pcDNA3/S+M	1	< 8	16
	2	< 8	16
	3	< 8	16
	4	< 8	16
	5	< 8	16
			
pcDNA3/S+M	1	< 8	16
	2	< 8	16
	3	< 8	16
	4	< 8	8
	5	< 8	8
			
pcDNA3	1	< 8	< 8
	2	< 8	< 8
	3	< 8	< 8
	4	< 8	< 8
	5	< 8	< 8

### eCTLA4 fusion strategy enhances CD8 T-cell responses

**Figure 3:**
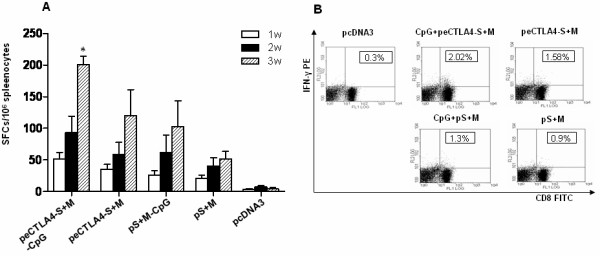
**HTNV N-specific cellular immune response following DNA vaccination**. (A) HTNV N-specific IFN-γ-producing CD8+ T cells demonstrated by ELISPOT Assay in groups of mice following DNA immunization. 4 mice of each group were euthanized on day 7, day 14 and day 21 after the first immunization. Splenocytes at 10^6 ^cells/well were tested in the presence of 10 μg/200 μl CTL epitope peptides M6. Number of spot forming cells (SFCs)/10^6 ^spleenocytes 2 was shown as mean plus standard deviation bar. SFCs in group of mice receiving pcDNA3/eCTLA4-S+M plasmids plus CpG motif was significantly higher than other groups (*p < 0.01) on day 21 after 1st immunization. (B) Detection of CD8+ CTL response specific for the epitope of hantavirus N protein by intracellular cytokine staining assay. One week after final boost, mice were euthanized. Splenocytes were restimulated with 10 μg/ml peptides (M6) for 5 h, and then stained with FITC-conjugated anti-CD8 and PE-conjugated anti-mouse IFN-γ McAbs. Mean frequencies of IFN-γ+ CD8+ cells in each group were shown in the right upper quadrants. Mean frequency of IFN-γ+ CD8+ cells in group of mice receiving pcDNA3/eCTLA4-S+M plasmids plus CpG motif was significantly higher than other groups (p < 0.05).

CD8+ T-cells play a vital role in protection against hantavirus infection by cell-mediated mechanisms. In order to evaluate the CD8+ T-cell response to vaccination, the splenocytes from mice vaccinated with DNA vaccine plasmids 1 week after each immunization were restimulated with HTNV N protein-specific peptides and analyzed by ELISPOT (Figure 3A). The splenocytes from mice 1 week after third immunization were restimulated and analyzed by Intracellular Cytokine Staining assay (Figure 3B). Number of CD8+IFN-γ-secreting splenocytes was significantly higher than other groups (p < 0.01) at 21 days after 1st immunization in mice receiving CpG+peCTLA4-M+S vaccine (Figure 3A). Consistently, mice vaccinated with pcDNA3/eCTLA4-S+M plasmids plus CpG1826 motif demonstrated higher frequencies of CD8+IFN+ T-cells to HTNV N protein-specific peptides compared with all the other groups in flow cytometery analysis. (Figure 3B). These results indicate that eCTLA4 fusion strategy could enhance the Th1-type cellular immune response.

## Discussion

DNA immunization with plasmids expressing hantavirus N protein and glycoprotein by intramuscular vaccination induced specific immune responses to the corresponding viral antigens in mice. In this study, we demonstrated that, a better magnitude of humoral and cellular immune responses could be generated in mice by DNA vaccine plasmids encoding HTNV N and GP fused to eCTLA4, a bioactive factor targeting to antigen presenting cells (APCs).

DNA vaccine has been demonstrated as a promising vaccination strategy for various viral infections [30]. Previous studies have shown good immunogenicity and protection efficacy of hantavirus DNA vaccine [6,8,31,32]. Hooper et al. demonstrated that DNA vaccination with a plasmid containing a cDNA representing the Seoul virus (SEOV) M segment elicited neutralizing antibody responses in mice and hamsters [6]. Gene gun vaccination with this DNA construct protected hamsters against infection with SEOV and HTNV [6,7]. They also reported a HTNV M gene-based DNA vaccine conferred sterile protection against infection in hamster model and elicited high levels of neutralizing antibodies in nonhuman primates [8]. Kamrud et al. also demonstrated a good immunogenicity of SEOV S gene-based DNA vaccine in hamster model [7]. Virus-neutralizing antibodies could be induced slightly in BALB/c mice following vaccination with DNA constructs encoding overlapped peptide fragments of Sin Nombre hantavirus (SNV) Gn and Gc protein [33]. However, these authors failed to reproduce the neutralizing antibody findings in a subsequent study with deer mouse model [34]. DNA vaccination with Puumala virus (PUUV) S segment also induced specific antibody response in mice [35]. Consistently, in our study, an N or GP-specific antibody response was detected respectively in mice after immunized with equal mixture of HTNV S gene and M gene-based DNA plasmids. A substantial level of neutralizing antibody was elicited by HTNV DNA vaccine. As cellular immune response also plays an important role in limiting virus infection and replication, we further evaluated the HTNV N-specific cellular immune response *in vitro*, and did see a high frequency of CD8+/IFN+ T-cells in mice receiving HTNV DNA vaccine.

It's generally accepted that modification of a viral antigen by fusion to a cellular protein, like eCTLA-4, could improve the efficacy of DNA vaccine[13,36-39]. Here we constructed DNA plasmids encoding HTNV N or GP fused to eCTLA-4 protein (pcDNA3/eCTLA4-S or M). Compared to DNA vaccine encoding HTNV N or GP alone, pcDNA3/eCTLA4-S (M) greatly improved the speed and magnitude of HTNV specific humoral immune response in mice. Lu et al. reported similar modulation effect of eCTLA4 on woodchuck hepatitis virus nucleoprotein in mice and woodchuck models [13]. Nicholas and his colleagues also observed the enhancement of immune responses to pro cathepsin B antigen in sheep model by fusion to eCTLA4 [40] In addition, there is a higher frequency of CD8+/IFN+ T-cells in mice immunized with pcDNA3/eCTLA4-S (M) DNA plasmids than that of pcDNA3/S (M). As the high affinity of eCTLA4 to its B7 ligand of APCs, our results indicated that eCTLA4 targeting may facilitate the antigen intake and processing by APCs, which will possibly improve the efficacy of DNA vaccine.

Another interesting finding of our study is that the efficacy of HTNV DNA vaccine was augmented by CpG motifs. When co-administration with CpG motifs, HTNV DNA vaccine induced better immune responses in mice compared with immunization with HTVN DNA vaccine alone. Vaccination with pcDNA3/eCTLA4-S (M) DNA plasmids plus CpG motifs elicited the highest antibody and cellular immune responses compared to all the other groups. Mice receiving pcDNA3/S (M) plus CpG motifs, though showed lower antibody titer one week after first immunization than that of mice vaccinated with pcDNA3/eCTLA4-S (M) DNA plasmids alone, exhibited comparable antibody response after the second injection. The recognition of CpG motifs is through toll-like receptor 9 (TLR-9) [41] and then induces a broad range of immunological effects on APCs [42]. Adjuvant effect of CpG motifs have been demonstrated in mice, humans as well as other species [14,15]. Thus, after co-delivery of CpG motifs with HTNV DNA vaccine, it's conceivable that APCs may be activated firstly by CpG motifs, then display enriched costimulatory molecules (including B7) on the surface. This early event may provide a more efficient intake of antigen mediated by eCTLA4 later on through binding with B7 ligand. This may, if any, at least partially explain the observed augmentation of humoral and cellular immune responses induced by HTNV DNA vaccine in combination with CpG motifs.

In summary, we have demonstrated that eCTLA4 fusion strategy could enhance antibody response and cellular immune response in mice generated by hantaan virus DNA vaccine. This adjuvant effect could be further augmented when co-delivery with CpG motifs. More work should be done to elucidate the mechanism of eCTLA4 fusion strategy. Overall, our results suggest that modification of viral antigen will be promising to confer efficient antigen presentation and improve the potency of DNA vaccine.

## Competing interests

The authors declare that they have no competing interests.

## References

[B1] Peters CJ Simpson GL Levy H Spectrum of hantavirus infection: hemorrhagic fever with renal syndrome and hantavirus pulmonary syndrome Annu Rev Med 1999 50 531 545 10.1146/annurev.med.50.1.531 10073292

[B2] Schmaljohn C Hjelle B Hantaviruses: a global disease problem Emerg Infect Dis 1997 3 95 104 10.3201/eid0302.970202 9204290 PMC2627612

[B3] Lee HW van der Groen G Hemorrhagic fever with renal syndrome Prog Med Virol 1989 36 62 102 2573914

[B4] Lee H Epidemiology and pathogenesis of hemorrhagic fever with renal syndrome The Bunyaviridae 1996 253 267

[B5] Hjelle B Vaccines against hantaviruses Expert Rev Vaccines 2002 1 373 384 10.1586/14760584.1.3.373 12901576

[B6] Hooper JW Kamrud KI Elgh F Custer D Schmaljohn CS DNA vaccination with hantavirus M segment elicits neutralizing antibodies and protects against seoul virus infection Virology 1999 255 269 278 10.1006/viro.1998.9586 10069952

[B7] Kamrud KI Hooper JW Elgh F Schmaljohn CS Comparison of the protective efficacy of naked DNA, DNA-based Sindbis replicon, and packaged Sindbis replicon vectors expressing Hantavirus structural genes in hamsters Virology 1999 263 209 219 10.1006/viro.1999.9961 10544095

[B8] Hooper JW Custer DM Thompson E Schmaljohn CS DNA vaccination with the Hantaan virus M gene protects Hamsters against three of four HFRS hantaviruses and elicits a high-titer neutralizing antibody response in Rhesus monkeys J Virol 2001 75 8469 8477 10.1128/JVI.75.18.8469-8477.2001 11507192 PMC115092

[B9] Boyle JS Brady JL Lew AM Enhanced responses to a DNA vaccine encoding a fusion antigen that is directed to sites of immune induction Nature 1998 392 408 411 10.1038/32932 9537327

[B10] Alegre ML Frauwirth KA Thompson CB T-cell regulation by CD28 and CTLA-4 Nat Rev Immunol 2001 1 220 228 10.1038/35105024 11905831

[B11] Thompson CB Allison JP The emerging role of CTLA-4 as an immune attenuator Immunity 1997 7 445 450 9354465 10.1016/s1074-7613(00)80366-0

[B12] Hueber AJ Matzkies FG Rahmeh M Manger B Kalden JR Nagel T CTLA-4 lacking the cytoplasmic domain costimulates IL-2 production in T-cell hybridomas Immunol Cell Biol 2006 84 51 58 10.1111/j.1440-1711.2005.01402.x 16405652

[B13] Lu M Isogawa M Xu Y Hilken G Immunization with the gene expressing woodchuck hepatitis virus nucleocapsid protein fused to cytotoxic-T-lymphocyte-associated antigen 4 leads to enhanced specific immune responses in mice and woodchucks J Virol 2005 79 6368 6376 10.1128/JVI.79.10.6368-6376.2005 15858020 PMC1091665

[B14] Mutwiri G Pontarollo R Babiuk S Griebel P van Drunen Littel-van den Hurk S Mena A Tsang C Alcon V Nichani A Ioannou X Gomis S Townsend H Hecker R Potter A Babiuk LA Biological activity of immunostimulatory CpG DNA motifs in domestic animals Vet Immunol Immunopathol 2003 91 89 103 10.1016/S0165-2427(02)00246-5 12543546

[B15] Klinman DM Currie D Gursel I Verthelyi D Use of CpG oligodeoxynucleotides as immune adjuvants Immunol Rev 2004 199 201 216 10.1111/j.0105-2896.2004.00148.x 15233736

[B16] Weeratna R Brazolot Millan CL Krieg AM Davis HL Reduction of antigen expression from DNA vaccines by coadministered oligodeoxynucleotides Antisense Nucleic Acid Drug Dev 1998 8 351 356 10.1089/oli.1.1998.8.351 9743472

[B17] Kojima Y Xin KQ Ooki T Hamajima K Oikawa T Shinoda K Ozaki T Hoshino Y Jounai N Nakazawa M Klinman D Okuda K Adjuvant effect of multi-CpG motifs on an HIV-1 DNA vaccine Vaccine 2002 20 2857 2865 10.1016/S0264-410X(02)00238-4 12126895

[B18] Schneeberger A Wagner C Zemann A Luhrs P Kutil R Goos M Stingl G Wagner SN CpG motifs are efficient adjuvants for DNA cancer vaccines J Invest Dermatol 2004 123 371 379 10.1111/j.0022-202X.2004.23208.x 15245438

[B19] Yang WS Bai XG Zhang WB Wang AL Bai XF Chen SY He YL Study on transmission of epidemic hemorrhagic fever virus via human placenta and localization in multi-organs of fetus Chinese journal of public health 1987 2 85 89

[B20] Liang M Li D Xiao SY Hang C Rossi CA Schmaljohn CS Antigenic and molecular characterization of hantavirus isolates from China Virus Res 1994 31 219 233 10.1016/0168-1702(94)90005-1 7909976

[B21] Xu FL Yang ZQ Yang CC Xiao SY Xiao H Wen L Serological characterization of a hantavirus from Hubei, China Acta Virol 2004 48 5 8 15230468

[B22] Liu Z Li D Li C Wang X Meng X Liang M Molecular cloning, nucleotides sequence and transient expression of S and M genome segment of hantavirus strain 84Fli Zhonghua Shi Yan He Lin Chuang Bing Du Xue Za Zhi 2002 16 48 51 11986746

[B23] Li C Liu F Liang M Zhang Q Wang X Wang T Li J Li D Hantavirus-like particles generated in CHO cells induce specific immune responses in C57BL/6 mice Vaccine 2010 28 4294 4300 10.1016/j.vaccine.2010.04.025 20433802

[B24] Bernstein DI Tepe ER Mester JC Arnold RL Stanberry LR Higgins T Effects of DNA immunization formulated with bupivacaine in murine and guinea pig models of genital herpes simplex virus infection Vaccine 1999 17 1964 1969 10.1016/S0264-410X(98)00469-1 10217595

[B25] Shimosato T Kitazawa H Katoh S Tohno M Iliev ID Nagasawa C Kimura T Kawai Y Saito T Augmentation of T(H)-1 type response by immunoactive AT oligonucleotide from lactic acid bacteria via Toll-like receptor 9 signaling Biochem Biophys Res Commun 2005 326 782 787 10.1016/j.bbrc.2004.11.119 15607737

[B26] Yoo YC Yoshimatsu K Koike Y Hatsuse R Yamanishi K Tanishita O Arikawa J Azuma I Adjuvant activity of muramyl dipeptide derivatives to enhance immunogenicity of a hantavirus-inactivated vaccine Vaccine 1998 16 216 224 10.1016/S0264-410X(97)00188-6 9607033

[B27] Zoller LG Yang S Gott P Bautz EK Darai G A novel mu-capture enzyme-linked immunosorbent assay based on recombinant proteins for sensitive and specific diagnosis of hemorrhagic fever with renal syndrome J Clin Microbiol 1993 31 1194 1199 8099085 10.1128/jcm.31.5.1194-1199.1993 PMC262902

[B28] Liang M Mahler M Koch J Ji Y Li D Schmaljohn C Bautz EK Generation of an HFRS patient-derived neutralizing recombinant antibody to Hantaan virus G1 protein and definition of the neutralizing domain J Med Virol 2003 69 99 107 10.1002/jmv.10259 12436484

[B29] Koch J Liang M Queitsch I Kraus AA Bautz EK Human recombinant neutralizing antibodies against hantaan virus G2 protein Virology 2003 308 64 73 10.1016/S0042-6822(02)00094-6 12706090

[B30] Davis HL McCluskie MJ DNA vaccines for viral diseases Microbes Infect 1999 1 7 21 10.1016/S1286-4579(99)80009-4 10594972

[B31] Custer DM Thompson E Schmaljohn CS Ksiazek TG Hooper JW Active and passive vaccination against hantavirus pulmonary syndrome with Andes virus M genome segment-based DNA vaccine J Virol 2003 77 9894 9905 10.1128/JVI.77.18.9894-9905.2003 12941899 PMC224585

[B32] Hooper JW Li D Vaccines against hantaviruses Curr Top Microbiol Immunol 2001 256 171 191 10.1007/978-3-642-56753-7_10 11217404

[B33] Bharadwaj M Lyons CR Wortman IA Hjelle B Intramuscular inoculation of Sin Nombre hantavirus cDNAs induces cellular and humoral immune responses in BALB/c mice Vaccine 1999 17 2836 2843 10.1016/S0264-410X(99)00096-1 10438054

[B34] Bharadwaj M Mirowsky K Ye C Botten J Masten B Yee J Lyons CR Hjelle B Genetic vaccines protect against Sin Nombre hantavirus challenge in the deer mouse (Peromyscus maniculatus) J Gen Virol 2002 83 Pt 7 1745 51 12075094 10.1099/0022-1317-83-7-1745

[B35] Koletzki D Schirmbeck R Lundkvist A Meisel H Kruger DH Ulrich R DNA vaccination of mice with a plasmid encoding Puumala hantavirus nucleocapsid protein mimics the B-cell response induced by virus infection J Biotechnol 2001 84 73 78 11035190 10.1016/s0168-1656(00)00329-1

[B36] Drew DR Boyle JS Lew AM Lightowlers MW Strugnell RA The human IgG3 hinge mediates the formation of antigen dimers that enhance humoral immune responses to DNA immunisation Vaccine 2001 19 4115 4120 10.1016/S0264-410X(01)00182-7 11457535

[B37] Kim SJ Lee C Lee SY Kim I Park JS Sasagawa T Ko JJ Park SE Oh YK Enhanced immunogenicity of human papillomavirus 16 L1 genetic vaccines fused to an ER-targeting secretory signal peptide and RANTES Gene Ther 2003 10 1268 1273 10.1038/sj.gt.3301997 12858192

[B38] Sailaja G Husain S Nayak BP Jabbar AM Long-term maintenance of gp120-specific immune responses by genetic vaccination with the HIV-1 envelope genes linked to the gene encoding Flt-3 ligand J Immunol 2003 170 2496 2507 12594275 10.4049/jimmunol.170.5.2496

[B39] Nayak BP Sailaja G Jabbar AM Enhancement of gp120-specific immune responses by genetic vaccination with the human immunodeficiency virus type 1 envelope gene fused to the gene coding for soluble CTLA4 J Virol 2003 77 10850 10861 10.1128/JVI.77.20.10850-10861.2003 14512535 PMC224956

[B40] Kennedy NJ Spithill TW Tennent J Wood PR Piedrafita D DNA vaccines in sheep: CTLA-4 mediated targeting and CpG motifs enhance immunogenicity in a DNA prime/protein boost strategy Vaccine 2006 24 970 979 10.1016/j.vaccine.2005.08.076 16242220

[B41] Hemmi H Takeuchi O Kawai T Kaisho T Sato S Sanjo H Matsumoto M Hoshino K Wagner H Takeda K Akira S A Toll-like receptor recognizes bacterial DNA Nature 2000 408 740 745 10.1038/35047123 11130078

[B42] Krieg AM CpG motifs in bacterial DNA and their immune effects Annu Rev Immunol 2002 20 709 760 10.1146/annurev.immunol.20.100301.064842 11861616

